# Membraneless and membrane-bound organelles in an anhydrobiotic cell line are protected from desiccation-induced damage

**DOI:** 10.1016/j.cstres.2024.04.002

**Published:** 2024-04-10

**Authors:** Clinton J. Belott, Oleg A. Gusev, Takahiro Kikawada, Michael A. Menze

**Affiliations:** 1 Department of Biology, University of Louisville, Louisville, KY, USA; 2 Division of Biotechnology, Institute of Agrobiological Sciences, National Agriculture and Food Research Organization, Tsukuba, Ibaraki, Japan; 3 Extreme Biology Laboratory, Institute of Fundamental Medicine and Biology, Kazan Federal University, Kazan, Tatarstan, Russia; 4 Molecular Biomimetics Group, Life Improvement by Future Technologies (LIFT) Center, Moscow, Russia; 5 Intractable Disease Research Center, Graduate School of Medicine, Juntendo University, Bunkyo-ku, Tokyo, Japan

**Keywords:** *Polypedilum vanderplanki*, Anhydrobiosis, Desiccation tolerance, Late embryogenesis abundant proteins, Membraneless organelles, Anhydrobiosis-related intrinsically disordered proteins

## Abstract

Anhydrobiotic species can survive virtually complete water loss by entering a reversible ametabolic glassy state that may persist for years in ambient conditions. The Pv11 cell line was derived from the egg mass of the anhydrobiotic midge, *Polypedilum vanderplanki*, and is currently the only available anhydrobiotic cell line. Our results demonstrate that the necessary preconditioning for Pv11 cells to enter anhydrobiosis causes autophagy and reduces mitochondrial respiration by over 70%. We speculate that reorganizing cellular bioenergetics to create and conserve energy stores may be valuable to successfully recover after rehydration. Furthermore, mitochondria in preconditioned cells lose their membrane potential during desiccation but rapidly restore it within 30 min upon rehydration, demonstrating that the inner mitochondrial membrane integrity is well-preserved. Strikingly, the nucleolus remains visible immediately upon rehydration in preconditioned cells while absent in control cells. In contrast, a preconditioning-induced membraneless organelle reformed after rehydration, demonstrating that membraneless organelles in Pv11 cells can be either stabilized or recovered. Staining the endoplasmic reticulum and the Golgi apparatus revealed that these organelles fragment during preconditioning. We hypothesize that this process reduces sheering stress caused by rapid changes in cellular volume during desiccation and rehydration. Additionally, preconditioning was found to cause the filamentous-actin (F-actin) network to disassemble significantly and reduce the fusion of adjacent plasma membranes. This study offers several exciting avenues for future studies in the animal model and Pv11 cell line that will further our understanding of anhydrobiosis and may lead to advancements in storing sensitive biologics at ambient temperatures for months or years.

## Introduction

The Pv11 cell line was derived from the egg mass of the midge *Polypedilum vanderplanki*, which is native to transient water pools in the semi-arid regions of sub-Saharan Africa. This remarkable animal's larvae can survive the dry season when water completely evaporates from the pools by drying and entering a reversible ametabolic glassy state (i.e*.,* anhydrobiosis). Desiccated larvae may persist in the anhydrobiotic state for several years until water becomes available.^1^

The Pv11 cell line is currently the only available anhydrobiotic cell line and can survive in the desiccated state at room temperature for at least 251 days.^2^ However, the cells require preconditioning in a 600 mM trehalose solution supplemented with 10% culture media for 48 h to enter anhydrobiosis successfully. Trehalose is a well-studied non-reducing disaccharide that several anhydrobiotic animals, including *P. vanderplanki* larva, accumulate to prepare for anhydrobiosis.^5^, ^6^, ^7^, ^8^, ^9^, ^10^, ^11^, ^12^, ^3^, ^4^ The molecular mechanisms by which trehalose is hypothesized to protect intracellular structures and proteins during desiccation include replacing water molecules with its hydroxyl groups, undergoing vitrification to limit molecular movement, and by a reduction in preferential binding of trehalose to newly exposed peptide groups in unfolding proteins.^13^ Recent work on Pv11 cells also detailed a novel sodium-ion dependent trehalose transporter capable of facilitating the release of intracellular Na^+^ ions with trehalose to more rapidly equalize the relatively hyperosmotic cytoplasm of the rehydrated cell with that of the culture media.^14^

How preconditioning, desiccation, and rehydration affect the morphology and physiology of organelles and other cellular structures in Pv11 cells is unexplored. This knowledge gap also includes the fate of constitutive membraneless organelles (MLOs), such as the nucleolus. MLOs are proteinaceous condensates that form because of the liquid–liquid phase separation (LLPS) of macromolecules. These proteinaceous condensates form target-selective, membraneless compartments that can be physiochemically significantly different from their surrounding milieu. For instance, examples of MLOs behaving more like organic solvents than aqueous solutions have been reported.^15^ In brief, proteinaceous LLPS typically occurs due to homotypic and heterotypic multivalent interactions between intrinsically disordered proteins or intrinsically disordered regions and often include nucleic acid partners. The resulting multivalent interactions favor some fraction of the protein separating from bulk water to form a dense phase. Intracellularly, the dense phase may act as an organelle and provide specialized functions. For detailed reviews on MLOs, please see.^16^, ^17^, ^18^, ^19^

The role of MLOs in anhydrobiosis is quickly gaining attention due to recent observations that anhydrobiosis-related intrinsically disordered (ARID) proteins from various anhydrobiotic plants and animals can undergo LLPS during desiccation or osmotic stress.^20^, ^21^, ^22^ Late embryogenesis abundant (LEA) proteins, originally named after their discovery in the late embryogenic stage of cotton (*Gossypium hirsutum*) seeds, are ARID proteins that can be found in both plants and animals.^23^, ^24^, ^25^ LEA proteins can be further subdivided into groups, of which three contain proteins that have been demonstrated to undergo LLPS. For this work, the grouping of LEA proteins refers to the Wise and Tunnacliffe nomenclature.^26^, ^27^ In animals, the group 6 LEA protein, *Afr*LEA6 from the brine shrimp *Artemia franciscana* forms a selective and domain-dependent MLO when ectopically expressed in *Drosophila melanogaster* Kc167 cells and can rapidly increase intracellular viscosity upon desiccation to resist rapid intracellular volume changes during desiccation.^29^, ^28^ LLPS was also observed with *Af*LEA1, a group 1 LEA protein from *A. franciscana* when ectopically expressed under similar conditions.^30^, ^31^, ^32^ In plants, three group 3 LEA proteins (LEA_4 Pfam group) were found to undergo LLPS.^20^ LEA9 from *Arabidopsis thaliana* is highly expressed in dry, mature seeds and displays osmotically regulated LLPS. At the same time, LEA48 homodimers and LEA42–LEA48 heterodimers were also found to undergo LLPS when transiently expressed in *Nicotiana benthamiana* (tobacco) leaves. Furthermore, the group 6 LEA protein Rab28 (Pfam 04927) in *Zea mays* was found to be selectively incorporated into the nucleolus in transgenic maize roots, which suggests that some LEA proteins may protect constitutive MLOs.^33^ Please see^21^, ^34^, ^35^ for further readings.

In addition to LEA proteins, eutardigrades contain unique ARID proteins with little to no sequence similarity to ARID proteins found in other anhydrobiotic species.^36^ Recently, exciting work on ARID proteins from *Ramazzottius varieornatus*, termed cytoplasmic abundant heat soluble (CAHS) proteins, revealed that CAHS3, CAHS8, and CAHS12 coalesce to reversibly form cytoskeletal-like filaments or condensates in response to hyperosmotic conditions when ectopically expressed in HEp-2 cells.^37^ Additionally, CAHS3 and CAHS12 filament formation was determined to be driven by a highly conserved C-terminal region. These results agree with another recent study that found CAHS8 from *Hypsibius exemplaris* also formed fibrils *in vitro*.^38^ Furthermore, CAHS1 from *R. varieornatus* was concurrently reported to undergo LLPS in hyperosmotic conditions when ectopically expressed in HeLa cells and formed fibrils *in vitro*.^39^ Like *Afr*LEA6, these CAHS proteins help buffer mechanical stress caused by water loss by intracellularly reinforcing the cell and causing it to adopt a more rigid morphology.

Since *P. vanderplanki* larvae and Pv11 cells express several group 3 LEA proteins in response to desiccation and preconditioning, respectively, it is reasonable to hypothesize that one or more of these proteins may have a role in the formation of stress-induced MLOs, play a role in the stabilization of constitutive MLOs, or cause the cell to adopt a more rigid morphology.^40^, ^41^, ^42^ In this study, we found that all studied cellular structures in Pv11 cells undergo significant morphological or physiological rearrangements during preconditioning, desiccation, and rehydration. Major findings include a 73% reduction of mitochondrial respiration during preconditioning and rapid recovery of the mitochondrial membrane potential after rehydration, the discovery of a preconditioning-induced MLO, and recovery of two different MLOs by either stabilization or reformation. Other findings of this study suggest that autophagy may aid in Pv11 cell anhydrobiosis, while fragmentation of the endoplasmic reticulum (ER) and Golgi apparatus fragmentation, disassembly of the F-actin, and reinforcement of the plasma membrane suggest that Pv11 cells adopt a more plastic morphology to buffer mechanical stress related to water loss and rehydration.

## Results

### Preconditioning causes autophagy, inhibits respiration, and protects the integrity of the inner mitochondrial membrane

**Fig. 1:**
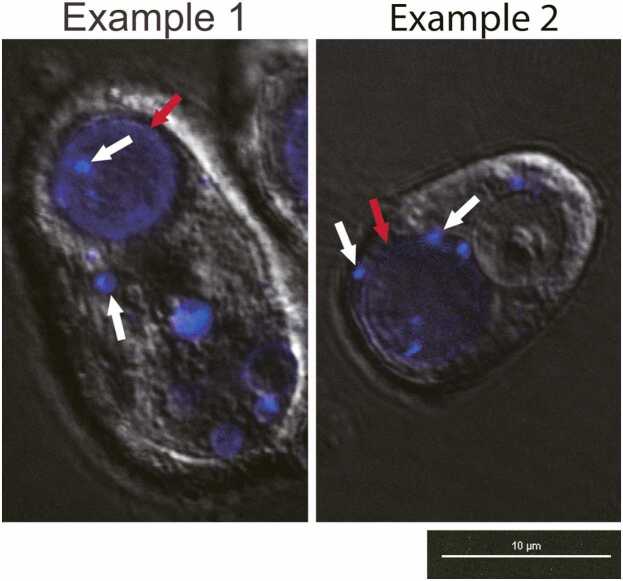
Preconditioning causes autophagy in Pv11 cells. LysoView 405 (blue) was used to stain acidic compartments in Pv11 after 48 h of preconditioning. Lysosomes (white arrows) can be observed before and after fusing into large autophagosomes (red arrows), demonstrating that preconditioning causes autophagy in Pv11 cells.

**Fig. 2:**
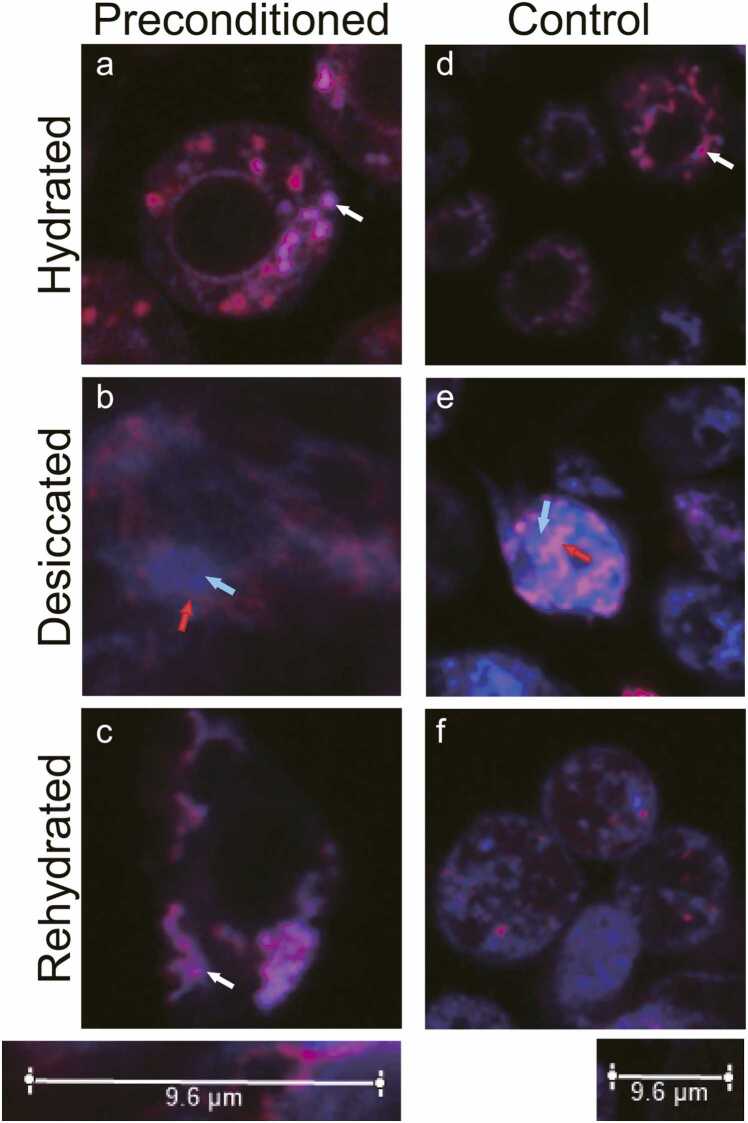
Preconditioning is required to protect the inner mitochondrial membrane and restore mitochondrial membrane potential after rehydration. Cells were stained with MitoView Blue (MVB) and Mitotracker Deep Red FM (DR). Both stains localize to the mitochondrion based on the mitochondrial membrane potential (a and d). DR is retained independently of the electrochemical potential in the mitochondria once localized unless the integrity of the inner mitochondrial membrane is compromised. In contrast, MVB will leak out of the mitochondria if the membrane potential is lost. White arrows indicate examples of the colocalization of DR and MVB. The inner mitochondrial membrane potential was lost after desiccation in control and preconditioned Pv11 cells, as indicated by MVB leaking out of the mitochondria (b and e). Blue arrows indicate examples of released MVB, and red arrows indicate examples of DR. Upon rehydration, mitochondria in preconditioned cells were still stained with DR, indicating that the integrity of the inner mitochondrial membrane was maintained (c). Furthermore, relocalization of MVB was observed after 30 min, demonstrating that the mitochondrial membrane potential was reestablished. Both DR and MVB staining were lost in control cells upon rehydration, suggesting that the integrity of the inner mitochondrial membrane was lost (f). Fluorescence intensities are not comparable among images. Different cells are viewed in each image. Figure S1 offers another example of MVB relocalization and recovery of cell morphology after 60 min of rehydration. Images of individual fluorescence channels are shown in Figure S2.

Preconditioned Pv11 cells were stained with LysoView 405 to stain acidic compartments. Bright blue lysosomes can be seen fusing into large autophagosomes (Figure 1). MitoView Blue (MVB; blue fluorescence) and MitoTracker Deep Red FM (DR; red fluorescence) were utilized to explore mitochondrial membrane potential changes and the integrity of the inner mitochondrial membrane during desiccation and rehydration in Pv11 cells (Figures 2 and S1). Both stains localize to the mitochondrion based on the membrane potential (Figure 2(a) and (d)). Once DR enters the organelle, it will react with thiol groups on mitochondrial matrix proteins and be retained regardless of whether the membrane potential is subsequently lost. If the integrity of the inner mitochondrial membrane is lost, DR–protein conjugates will leak from the mitochondria, resulting in nonspecific DR staining. In contrast, MVB is not retained after the mitochondrial membrane potential is lost but will reenter the organelle if the membrane potential has been restored. In control and preconditioned samples, the mitochondrial membrane potential is lost upon desiccation (i.e., loss of MVB colocalization with DR) (Figure 2(b) and (e)). However, the mitochondrial membrane potential reestablishes (i.e., relocalization of MVB) within 30 min after rehydration in preconditioned cells (Figure 2(c)). While DR staining was well-preserved in viable preconditioned cells after desiccation and rehydration, it was primarily diffused throughout control cells (Figure 2(f)). In addition, preconditioned cells with intact mitochondria were reattached to the imaging plate within 1 h after rehydration (Figure S2).

**Fig. 3:**
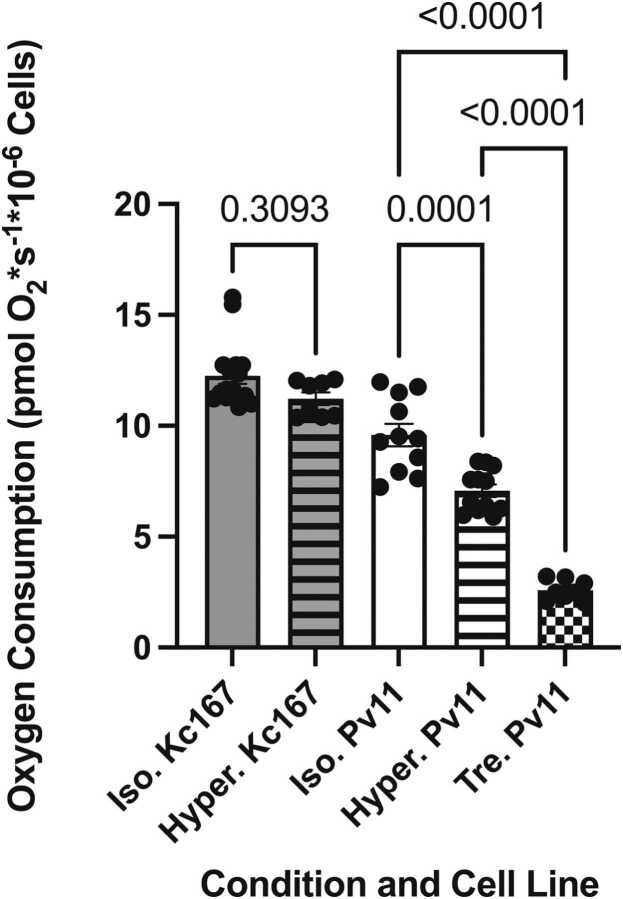
Preconditioning significantly reduces mitochondrial respiration in Pv11 cells by 73%. In addition, a significant decrease in respiration was observed in Pv11 cells when subjected to acute hyperosmotic (+200 mM NaCl) stress. Kc167 cells (*Drosophila melanogaster*) are sensitive to water loss and were used as a reference for the acute hyperosmotic response observed in Pv11 cells. Respiration in Kc167 did not significantly decrease in response to the acute 200 mM NaCl stress, suggesting that the decrease in respiration observed in Pv11 cells was regulatory and not due to cellular or mitochondrial damage. Kc167 cells did not survive preconditioning and could not be compared to the response observed in preconditioned Pv11 cells. The *P* value for each comparison is listed (one-way analysis of variance (ANOVA) with Tukey post-hoc analysis, *N* = 8–16). Error bars represent the standard error of the mean.

Cellular respirometry revealed a 73% decrease in the oxygen consumption rate of preconditioned Pv11 cells (Figure 3). Desiccation-sensitive *D. melanogaster* Kc167 cells and Pv11 cells were acutely subjected to culture media supplemented with 200 mM NaCl to gain insights into if this reduction in respiration in Pv11 cells was due to regulation or the result of unspecific cellular or mitochondrial damage. The hypertonic media did not significantly decrease the respiration of Kc167 (*P* value = 0.309). In contrast, hypertonicity significantly decreased respiration in Pv11 cells by 26% (*P* value = 0.0001). Kc167 cells did not survive preconditioning and could not be compared to preconditioned Pv11 cells.

### Preconditioning stabilizes the nucleolus, while a preconditioning-induced MLO in the cytoplasm reassembles upon rehydration

**Fig. 4:**
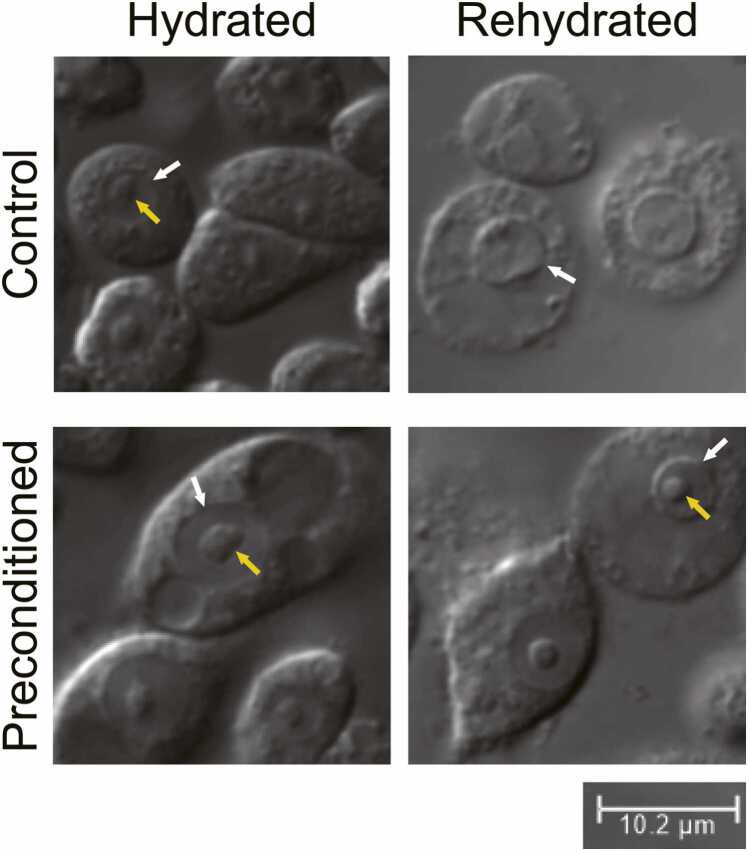
The nucleolus is stabilized by preconditioning. The nucleus (white arrow) and nucleolus (yellow arrow) were visible in most preconditioned cells, regardless of cell viability. In contrast, the nucleolus was virtually absent in all control cells, even when the nuclear envelope was visible. These data demonstrate that the nucleolus, a membraneless organelle, can maintain its dense phase throughout desiccation and rehydration. The images shown are magnified examples of cells from a representative image of each condition. Please see Figure S2 for the representative images and quantification of visible nucleoli.

**Fig. 5:**
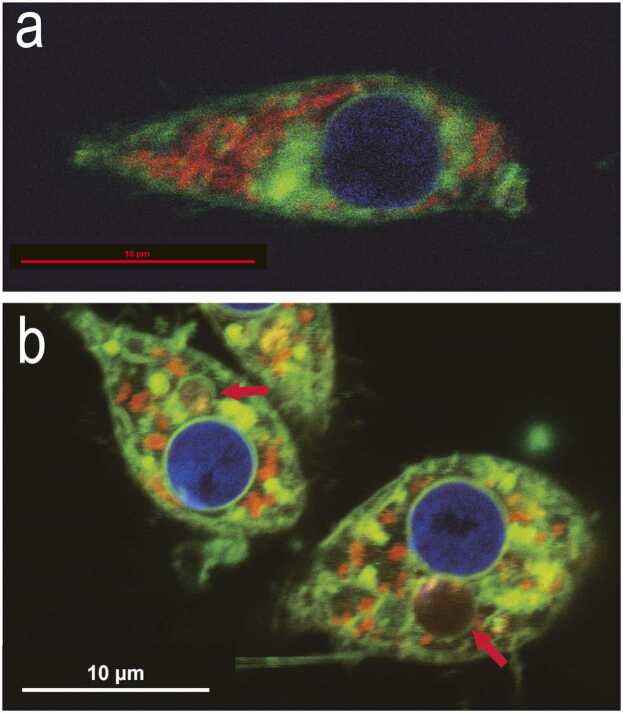
Preconditioning causes the ER and Golgi apparatus to fragment and the formation of a preconditioning-induced MLO. The ER/Golgi Cytopainter staining kit was used to stain the ER (orange), Golgi apparatus (green), and nucleus (blue) in control (a) and preconditioned Pv11 cells (b). The red ER stain appears orange in merged images due to slight off-staining from the green Golgi apparatus stain. Unexpectedly, the staining kit also stained a preconditioning-induced MLO (red; red arrows) in preconditioned Pv11 cells. This compartment accumulated the red ER stain but excluded the green Golgi apparatus stain. It is unknown why the ER stain accumulated and the Golgi apparatus stain was excluded. Fluorescence intensities are not comparable between images. Both scale bars represent 10 µm. Please see Figures 6 and S5 for the coalescence of the preconditioning-induced MLO during rehydration. Abbreviations used: ER, endoplasmic reticulum; MLO, membraneless organelle.

**Fig. 6:**
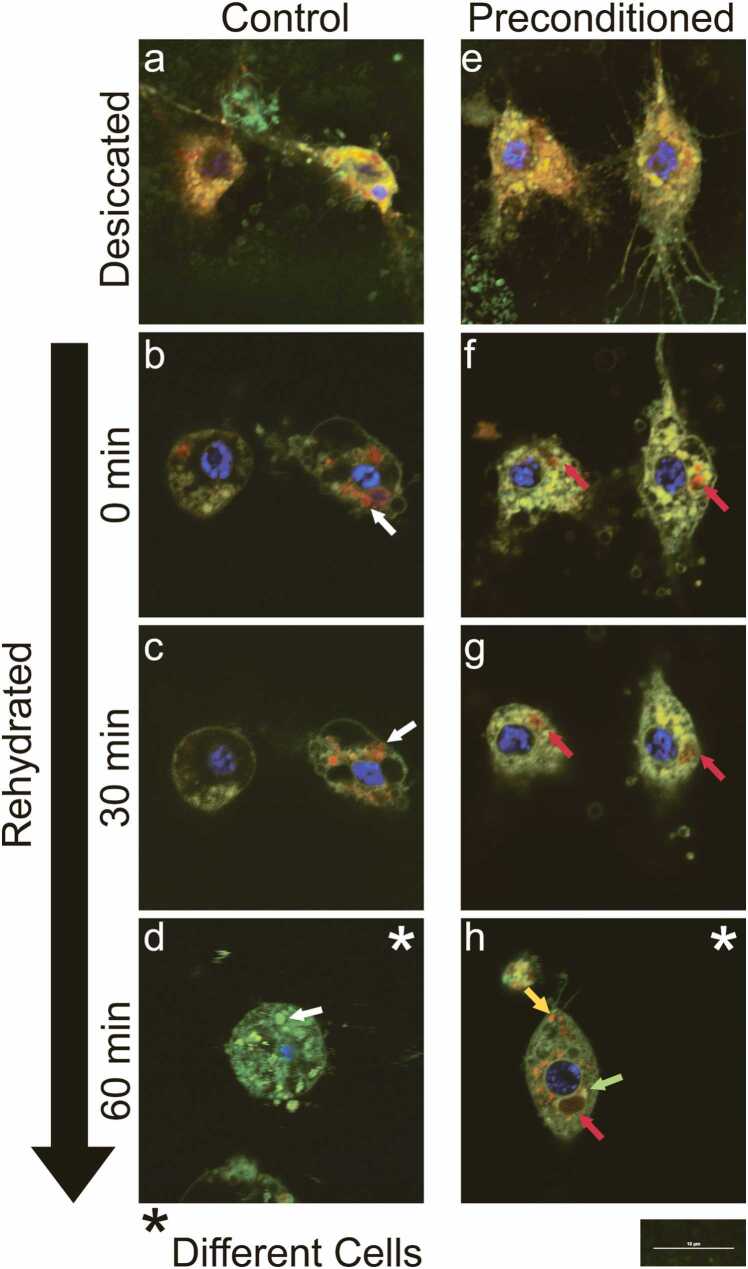
Preconditioning is required to recover the ER and Golgi apparatus after rehydration and maintain nuclear envelope integrity. The ER/Golgi Cytopainter staining kit was used to stain the ER (orange; orange arrow), Golgi apparatus (green; green arrow), nucleus (blue), and the preconditioning-induced MLO (red; red arrow). The red ER stain appears orange in merged images due to slight off-staining from the green Golgi apparatus stain. Upon desiccation, the ER and Golgi apparatus stains loose localization in control and preconditioned cells, and the preconditioning-induced MLO disassembles (a and e). After rehydration, the orange and green staining observed in control cells (white arrows are examples) results from unspecific staining due to loss of cellular organization (b and c). In preconditioned cells, the preconditioning-induced MLO rapidly reassembles within 30 min and progressively excludes the green Golgi apparatus stain (f and g). After 60 min, loss of cellular organization continues in control cells (d). In contrast, recovered ER and Golgi apparatus fragments can be observed in preconditioned cells, and the nuclear envelope is maintained (h). Additionally, the preconditioning-induced MLO reforms its predesiccated morphology and completely excludes the green Golgi apparatus stain. Fluorescence intensities are not comparable among images. The scale bar represents 10 µm. Images of individual fluorescence channels are shown in Figure S5. Abbreviations used: ER, endoplasmic reticulum; MLO, membraneless organelle.

The nucleolus, likely the most complex MLO in the cell, was observed before desiccation in both control and preconditioned cells (Figure 4). The nucleolus was visible immediately upon rehydration in most preconditioned cells, regardless of their viability, and virtually absent in control cells. Quantification of a representative image from each condition yielded a visible nucleolus in 87% of preconditioned Pv11 cells and 6% of control cells (Figure S3). In addition, a preconditioning-induced MLO was discovered to assemble in preconditioned Pv11 cells (Figure 5). The MLO displayed red fluorescence due to off-staining from the red ER stain and the exclusion of the green Golgi stain. This structure was confirmed to be an MLO based on its disassociation during desiccation, formation of separate spherical structures during rehydration, the subsequent coalescing of these structures, and the progressive exclusion of the green Golgi apparatus stain (Figures 6 and S5). At the time of experimentation, the manufacturer of the Cytopainter staining kit reported that they did not know how the Endoplasmic reticulum (ER) stain specifically targets the ER and did not disclose any information about the structure of the stains, therefore why the ER stain accumulated in the MLO while the Golgi apparatus stain was excluded remains unknown.

### Preconditioning causes ER and Golgi apparatus fragmentation and protects the ER, Golgi apparatus, and nuclear envelope during desiccation and rehydration

In Pv11 cells grown in culture media, the ER forms extended networks, while the Golgi apparatus can be viewed primarily between the ER network and nucleus (Figure 5(a)). In addition, the ER often spans long axon-like structures at one or both polar ends of the cell that are accompanied by Golgi outposts (Figure S4). In contrast, preconditioning causes the ER and Golgi apparatus fragmentation (Figure 5(b)). In control cells, the ER and Golgi apparatus were not recovered (Figures 6(a)–(d) and S5). However, these organelles were recovered within 60 min after rehydration in preconditioned cells (Figures 6(e)–(h) and S5). In addition, preconditioned cells also maintained nuclear envelope integrity upon rehydration, which was lost in control cells, depicted by the green Golgi apparatus stain diffusing into the nucleus of control cells.

### Preconditioning promotes F-actin network disassembly and prevents plasma membrane fusion but does not increase intracellular viscosity during desiccation

**Fig. 7:**
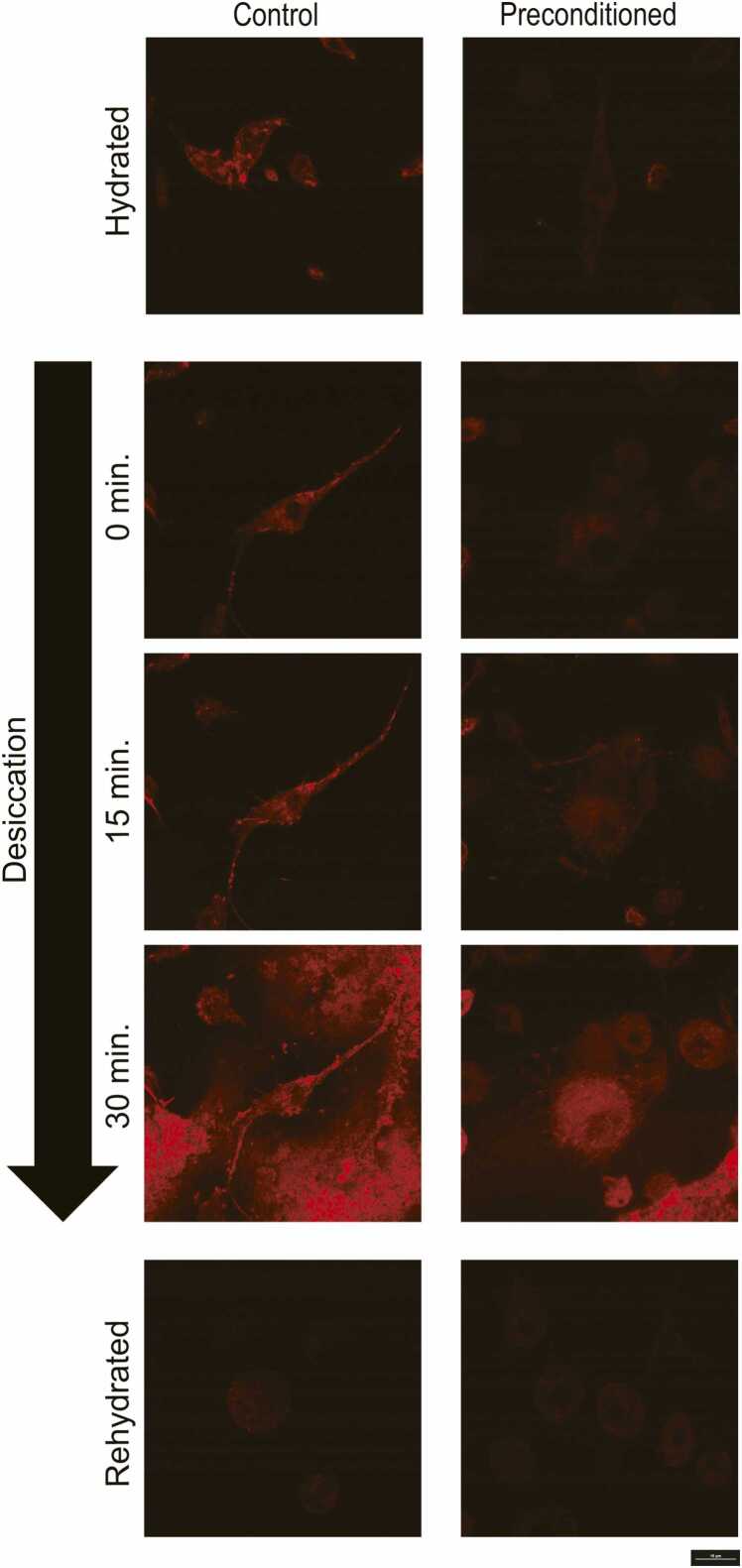
Preconditioning causes the filamentous actin (F-actin) network (red) to disassemble significantly. CellMask Orange Actin Tracking Stain was used to stain the F-actin network in control and preconditioned Pv11 cells. This stain does not stain unpolymerized globular actin (G-actin). Fluorescence intensities are comparable among all images. The increase in fluorescence intensity around cells and in the cytosol observed for both conditions at 30 min is representative of the stain precipitating during desiccation. The decrease in F-actin in preconditioned cells is hypothesized to aid in intracellular volume changes during desiccation and rehydration. The scale bar represents 10 µm. G-actin; Globular actin.

**Fig. 8:**
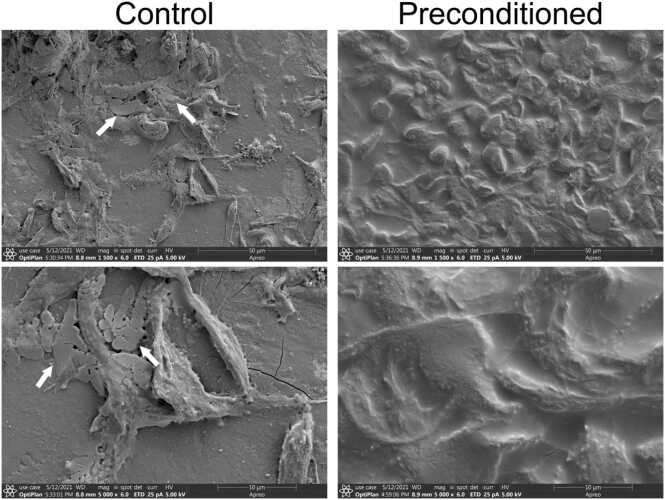
Preconditioning prevents plasma membrane fusion among Pv11 cells during desiccation. The top images offer a broad view of control and preconditioned samples (scale bars = 50 µm), while the bottom images are focused (scale bars = 10 µm). Membrane fusion was observed in control cells (white arrows indicate examples) and not for preconditioned cells. Control and preconditioned cells were resuspended in Dulbecco's phosphate-buffered saline (DPBS) before plating and drying to remove bulk extracellular trehalose in the preconditioned sample. DPBS; Dulbecco’s phosphate-buffered saline.

The F-actin network was stained in preconditioned and control Pv11 cells (Figure 7). Importantly, this stain does not target globular actin (unpolymerized actin). The F-actin network can be seen in the cell body and traveling along the axon-like structures in control cells. However, little or no F-actin was visible in preconditioned cells. After 30 min of desiccation, both samples showed a sharp increase in red fluorescence, likely due to precipitating stains. After rehydration, F-actin was not visible in control or preconditioned cells. In addition, Nile Red staining revealed no significant increase in intracellular viscosity between control and preconditioned Pv11 cells during drying (Figure S6). Despite a decrease in the assembly of the F-actin network and lack of increasing intracellular viscosity to compensate for the loss in structural rigidity, scanning electron microscopy (SEM) revealed that preconditioning nevertheless significantly reduced plasma membrane fusion among cells during desiccation (Figure 8).

## Discussion

This study expands our understanding of how adaptation for anhydrobiosis requires reorganizing intracellular structures during preconditioning, desiccation, and rehydration by comparing morphological and physiological changes in preconditioned and nonpreconditioned Pv11 cells, as well as water-stress-sensitive cells. We hypothesize that autophagy is not only a response to the low-nutrient preconditioning media but may play a role in the anhydrobiosis of Pv11 cells by removing unnecessary or desiccation-sensitive proteins to fine-tune the proteome for desiccation, rehydration, and overall recovery. Preconditioning also reduced mitochondrial respiration by a striking 73%, likely contributing to an overall reduction in reactive oxygen species (ROS) formation. Mitochondria in preconditioned cells were also able to reestablish their membrane potential within 30 min after rehydration, demonstrating that preconditioning protects the integrity of the inner mitochondrial membrane. Furthermore, preconditioning stabilized the nucleolus in its dense phase. In contrast, the discovered preconditioning-induced MLO dissociates upon desiccation and rapidly reforms upon rehydration. Together, these data demonstrate the MLOs in Pv11 cells may be recovered by either stabilization during desiccation or reassembly after rehydration. Lastly, preconditioning caused the ER and Golgi apparatus networks to fragment, along with a significant dissociation of the F-actin network. We hypothesize that these reorganizations allow the Pv11 cells to tolerate rapid cell volume changes by adopting a more overall plastic (i.e., less ridged) morphology. This hypothesis was further supported by the observation that preconditioned Pv11 cells do not undergo a significant increase in intracellular viscosity during desiccation compared to control cells. Lastly, the plasma membranes of preconditioned cells were found to undergo significantly less fusion among adjacent cells during desiccation than controls, demonstrating that resisting rapid volume change due to desiccation and rehydration was not necessary to preserve plasma membrane integrity. These characterizations yield several exciting avenues for future studies that may lead to biotechnological advancements in storing sensitive biological materials, including protein condensates, in a desiccated state for prolonged periods of time in ambient conditions. These applications have already been discussed elsewhere.^35^

Pv11 cells require preconditioning for 48 h in a solution containing 10% culture media and 600 mM trehalose to achieve anhydrobiosis.^2^ Unsurprisingly, the low-nutrient preconditioning media causes autophagosomes to form, identified *via* lysosome staining and the visualization of lysosomes located both inside and outside of the autophagosomes.^43^ While it could be presumed that the low-nutrient content of the preconditioning media is the underlining cause of autophagy in Pv11 cells, trehalose accumulation could potentially play a role in autophagy initiation or regulation, as was the case in the resurrection grass *Tripogon loliiformis*.^44^ Indirect evidence for this was presented in a recent report where trehalose and heat shock factor 1, a central regulator of anhydrobiosis-related genes in *P. vanderplanki*, were both required for Pv11 cells to upregulate several genes related to autophagy and anhydrobiosis (e.g., LEA proteins).^45^, ^46^ As previously suggested for the yeast *Saccharomyces cerevisiae* and *T. loliiformis*, autophagy may improve desiccation tolerance by removing desiccation-sensitive or unnecessary proteins before desiccation while increasing crucial energy stores for recovery.^47^, ^44^

The danger ROS pose during desiccation is well-established, and many anhydrobiotic species, including *P. vanderplanki*, upregulate their antioxidant systems and enter a state of metabolic arrest that contributes to limiting ROS production.^21^, ^40^, ^46^, ^48^, ^49^, ^50^, ^51^, ^52^, ^53^, ^54^, ^55^, ^56^, ^57^, ^58^, ^59^, ^60^, ^61^ Preconditioned Pv11 cells showed a striking 73% decrease in mitochondrial respiration compared to control cells. This significant reduction in cellular respiration likely contributes to an overall decline in ROS production in preconditioned Pv11 cells during desiccation and rehydration. In addition, it was discovered that mitochondria in Pv11 cells rapidly reduce respiration by 26% when acutely challenged with culture media supplemented with 200 mM NaCl to induce hyperosmotic stress. As a comparative model, desiccation-sensitive Kc167 cells derived from the embryo mass of *D. melanogaster*, a nonanhydrobiotic species also belonging to the Diptera taxonomic order, were used. Kc167 cells did not show a significant decrease in respiration when challenged with the same acute hyperosmotic stress, suggesting that inhibition of respiration in Pv11 cells is due to metabolic downregulation and not organelle damage during preconditioning. Furthermore, the integrity of the inner mitochondria membrane of preconditioned Pv11 cells was maintained through desiccation and rehydration, and remarkably, mitochondria were able to reestablish their membrane potential within 30 min following rehydration. These results confirm metabolomic data from anhydrobiotic *P. vanderplanki* larvae, where citrate accumulation during desiccation was hypothesized to enable a rapid restart of the citric acid cycle and mitochondrial respiration upon rehydration.^58^

While imaging mitochondria in Pv11 cells, it was discovered that preconditioning stabilizes the nucleolus throughout desiccation and rehydration. In the representative images, the nucleolus was visible in 87% of preconditioned Pv11 cells, regardless of cell viability, and did not require reassembly during rehydration (i.e., it remained in its dense phase). In contrast, the nucleolus was only visible in 6% of control cells with a visible nuclear envelope, suggesting that the physicochemical properties of the nucleolus or the nucleoplasm change due to preconditioning. Since trehalose can freely pass through nuclear pores and several LEA proteins from *P. vanderplanki* are known to localize to the nucleus, it is reasonable to hypothesize that these protectants play a significant role in stabilizing the nucleolus during desiccation and rehydration.^41^, ^62^ The nucleolus is a MLO that functions as the catalytic center for ribosome biogenesis and consists of three distinct phases (i.e., subcompartments): a fibril center, a surrounding dense fibrillar component, and a peripheral granular component (for review, please see^63^). Given the essential biological roles of the nucleolus, protecting this organelle from irreversible dissociation or aggregation damage during desiccation and rehydration is critical. However, the nucleolus is a complex organelle, and the molecular mechanisms by which preconditioned Pv11 cells preserve their nucleolus throughout desiccation and rehydration are likely complex and involve multiple protective agents that may differ for each subcompartment. In contrast to the nucleolus, the discovered preconditioning-induced MLO rapidly reassembles within 30 min, although its composition is unknown. Nevertheless, these data demonstrate that Pv11 cells can recover MLOs by either stabilization or reformation. Considering that irreversible phase transitions lead to several pathological disorders, including Alzheimer's disease, the molecular mechanisms employed by Pv11 cells to protect their MLOs may be of interest to emerging industries focused on treating pathological condensates.^64^, ^66^, ^65^

In addition to mitochondria, morphological changes in the ER, Golgi apparatus, and nucleus were also explored. The tissue from which the Pv11 cell line was derived is unknown and may be neuronal in origin based on the axon-like structures that contain an elongated ER network and Golgi outposts, all of which are typical of neurons.^67^, ^42^ During preconditioning, however, these networks fragment. We hypothesize that network fragmentation may reduce sheer stress on these organelles as cell volume rapidly changes during desiccation and rehydration. An alternative but not mutually exclusive hypothesis is that fragmentation may allow endogenous LEA proteins and other protectants, such as trehalose, to protect against desiccation-induced membrane rigidification more effectively, fusion, and nonlamellar phase transitions.^35^ In addition to recovering the ER and Golgi apparatus from a fragmented state, the integrity of the nuclear envelope was maintained after rehydration in preconditioned cells but not in control cells, demonstrating that preconditioning is also required to protect the nuclear envelope.

The F-actin network, intracellular viscosity, and plasma membrane structural integrity were explored by building on the hypothesis that ER and Golgi fragmentation may be related to tolerating rapid cell volume changes during desiccation and rehydration. The F-actin network in preconditioned cells was found to be significantly disassembled after preconditioning, suggesting that preconditioned Pv11 cells relax the rigidity of their cytoskeletal network. Furthermore, Nile Red staining revealed no significant intracellular viscosity changes between control and preconditioned Pv11 cells during desiccation. This result suggests that they do not increase intracellular viscosity to resist rapid volume changes during desiccation and rehydration.^68^, ^69^, ^28^, ^70^, ^71^ Nevertheless, Scanning electron microscopy data of desiccated Pv11 cells revealed that preconditioning significantly reduced plasma membrane fusion among adjacent cells. Altogether, these data support the hypothesis that preconditioning causes Pv11 cells to enter a more plastic (i.e., less ridged) state to tolerate rapid changes in cell volume during desiccation and rehydration. These results contrast with observations for *Afr*LEA6 from *A. franciscana* and CAHS proteins from *R. varieornatus*, where *Afr*LEA6 was found to increase the intracellular viscosity during desiccation rapidly, and CAHS proteins were found to coalesce and form cytoskeletal-like filaments during hyperosmotic stress. ARID proteins underwent a phase transition in both instances to resist intracellular volume changes during water loss. For *Afr*LEA6, this was shown to reduce plasma membrane fusion of adjacent cells during desiccation significantly. However, protection of the plasma membrane in Pv11 cells may perhaps be attributed to PvLEA1 and three LEA-like proteins (PvLIL1, PvLIL2, and PvLIL10), all of which localize to the plasma membrane and are upregulated during preconditioning in Pv11 cells and during desiccation in the anhydrobiotic larvae.^46^, ^62^, ^72^ Considering that trehalose has been shown to protect lipid bilayers of various compositions against desiccation-induced damage by itself and in concert with ARID proteins (*Afr*LEA2 and *Afr*LEA3m) from *A. franciscan*a, it is highly likely that trehalose also significantly contributes to the protection of the plasma membrane and other lipid bilayers in Pv11 cells.^73^ Lastly, larvae of *P. vanderplanki* are considerably larger than any other anhydrobiotic animal, reaching up to 7 mm in length. Therefore, cell shrinkage during desiccation may be necessary to prevent damage at the tissue level, as water also leaves extracellular spaces.

Much remains to be understood about how intracellular structures in Pv11 cells reorganize to prepare for and recover from desiccation. This work provides several exciting avenues for future studies in the animal model and the Pv11 cell line. Understanding how organelles are protected in *P. vanderplanki* larvae and Pv11 cells will further our understanding of the molecular mechanisms governing anhydrobiosis and may lead to advancements in developing technology to reliably store sensitive biologics in the desiccated state for months or even years at ambient temperatures.

## Methods

### Cell culture and preconditioning of Pv11cells

Pv11 cells were derived from embryos of *P. vanderplanki* from Nigeria and are still being cultured at the Institute of Agrobiological Sciences, National Agriculture and Food Organization, Tsukuba, Japan.^74^ *D. melanogaster* Kc167 cells were purchased from the Drosophila Genomics Research Center (Stock1; https://dgrc.bio.indiana.edu//stock/1; RRID:CVCL_Z834; Bloomington, IN, USA).^75^ Both cell lines were cultured at 25 °C in IPL-41 media (Gibco, Waltham, MA, USA), supplemented with 10% fetal bovine serum, heat-inactivated at 55 °C for 30 min (Atlanta Biologicals, Flowery Branch, GA, USA) and 2.6 g/L tryptone phosphate broth (VWR, Radnor, PA, USA). Pv11 cells were preconditioned by resuspending 3–4 × 10^6^ cells/mL in a 600 mM trehalose (Pfanstiehl, Inc., Waukegan, IL, USA) solution supplemented with 10% culture media for 48 h at 25 °C. Kc167 cells did not survive preconditioning.

### Confocal microscopy

Images were taken on either a TCS SP8 Leica confocal microscope (Leica Microsystems, Shinjuku City, Japan) or a Nikon A1R confocal microscope (Nikon Instruments Inc., Melville, NY) with a 100× oil immersion objective. Laser excitation/emission wavelengths were as follows: blue fluorescence = 405/450 nm, green fluorescence = 488/525 nm, and red fluorescence = 552 (Leica) or 561 (Nikon)/595 nm. To precondition Pv11 cells for confocal microscopy, 1.5–2 × 10^6^ cells/mL were plated in 500 µL/chambers (working volume) of a 4-compartment, 35 mm CELLview cell culture dish (Greiner Bio-One, Kremsmunster, Austria). All stained samples were exposed to the respective dye for 10 min at 25 °C, carefully washed with 200 µL Dulbecco's phosphate-buffered saline (DPBS), and imaged in 200 µL of DPBS. Dyes used were LysoView 405 (Biotium, Fremont, CA, USA), Golgi/ER Cytopainter staining kit (Abcam, Boston, MA, USA), MVB (Biotium), and MitoTracker DR (Thermo Fisher Scientific, Waltham, MA, USA), 9-diethylamino-5H-benzo[*a*]phenoxazin-5-one (Nile Red; TCI, Fremont, CA, USA), and CellMask Orange Actin Tracking Stain (Invitrogen, Carlsbad, CA, USA). The LysoView 405, Golgi/ER Cytopainter staining kit and CellMask Orange stains were prepared according to their manufacturer's instructions. MVB and MitoTracker Deep Red had final working concentrations of 100 nM and 250 nM in DPBS, respectively. Nile Red was prepared and used as previously described, with a working concentration of 0.1 µg/mL.^28^ Sample desiccation was achieved by carefully removing all remaining DPBS and ambient air drying of the samples. In all cases, samples were rehydrated by gently adding 200 µL DPBS into the cell chamber.

### Scanning electron microscopy (SEM)

A Zeiss Supra 35VP (Carl Zeiss, White Plains, NY, USA) was used for Scanning electron microscopy imaging. Twenty microliter of control or preconditioned Pv11 cells resuspended in DPBS at a concentration of 3–4 × 10^6^ cells/mL were plated on aluminum stages and allowed to settle for 30 min. Afterward, excess media was carefully removed, and cells were desiccated at 0% relative humidity in a sealed desiccation chamber over anhydrous calcium sulfate (Drierite; Xenia, OH, USA) for 2–3 days at 25 °C. Samples were then sputter coated with 19.8 nm gold and immediately imaged.

### High-resolution respirometry

Oxygen flux was measured using an Oxygraph-2k high-resolution respirometer (Oroboros Instruments, Innsbruck, Austria). For all tested conditions, cells were resuspended to a concentration of 2.5 × 10^6^ cells/mL in their respective media, and respiration rates were measured at 25 °C. Control samples were resuspended in culture media, while cells subjected to acute hyperosmotic stress were resuspended in culture media supplemented with 200 mM NaCl (VWR). Respiration rates of preconditioned Pv11 cells were measured in preconditioning media. Statistics were performed on Prism (one-way analysis of variance (ANOVA) with Tukey post-hoc analysis, *N* = 8–16, *F* = 95.70; Brown-Forsythe Test *P* value = 0.1439; GraphPad Software, San Diego, CA, USA).

## Declarations of interest

The authors declare the following financial interests/personal relationships which may be considered as potential competing interests: Clinton J. Belott reports financial support was provided by Japan Society for the Promotion of Science. Clinton J. Belott reports financial support was provided by National Science Foundation. If there are other authors, they declare that they have no known competing financial interests or personal relationships that could have appeared to influence the work reported in this paper.

## Data Availability

Data will be made available on request.
