# Supplementary material for: Membraneless and membrane-bound organelles in an anhydrobiotic cell line are protected from desiccation-induced damage
Source: Cell Stress Chaperones. 2024 Apr 10;29(3):425–36. doi: 10.1016/j.cstres.2024.04.002 (PMC11061232; doi:10.1016/j.cstres.2024.04.002)
Supplement: Supplementary file 1 — Supplementary material [file mmc1.pdf]

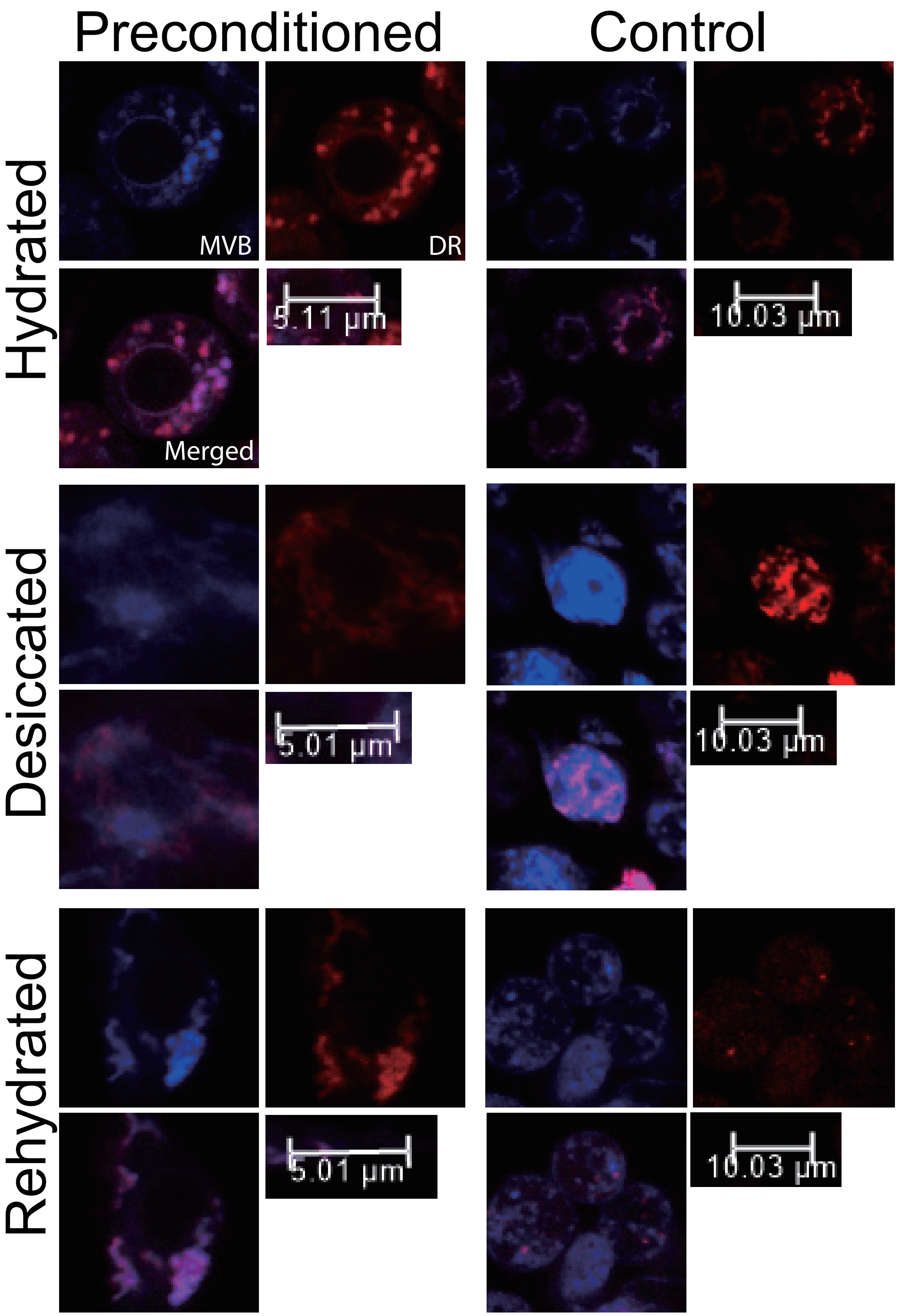

Fig. S1. Individual fluorescence channels for Fig. 2. Cells were stained with MitoView Blue (MVB) and Mitotracker Deep Red FM (DR). Both stains localize to the mitochondria based on the mitochondria membrane potential. DR is retained in the mitochondria once localized unless the integrity of the inner mitochondrial membrane is compromised. In contrast, MVB will leak out of the mitochondria if the membrane potential is lost. The inner mitochondrial membrane potential was lost after desiccation in preconditioned Pv11 cells, as indicated by MVB leaking out of the mitochondria. Upon rehydration, mitochondria in preconditioned cells were still stained with DR, indicating that the integrity of the inner mitochondrial membrane was maintained. Furthermore, relocalization of MVB was observed after 30 min, demonstrating that the mitochondrial membrane potential was reestablished. Fluorescence intensities are not comparable among images, and different cells are viewed in each image. Please see Fig. 2 for larger merged images.

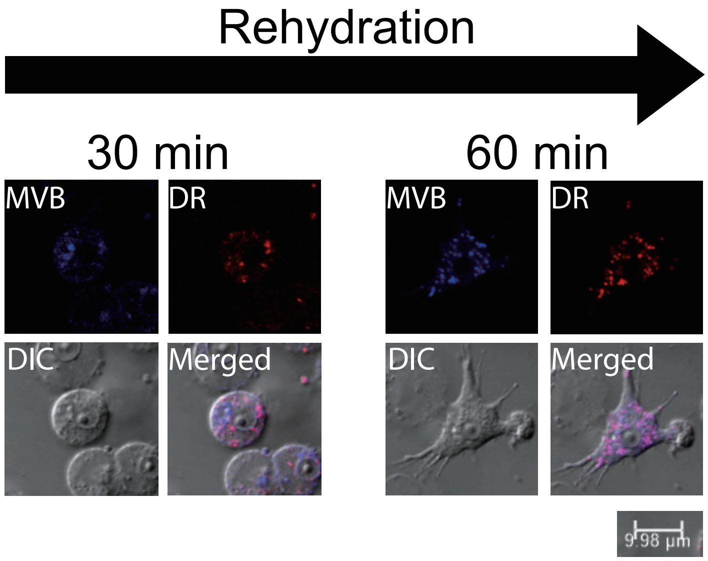

Fig. S2. Preconditioning for 48 h is required for Pv11 cells to enter and exit anhydrobiosis successfully. Cells were stained with MitoView Blue (MVB) and Mitotracker Deep Red FM (DR). Both stains localize to the mitochondria based on the mitochondria membrane potential. DR is retained in the mitochondria once localized unless the integrity of the inner mitochondrial membrane is compromised. In contrast, MVB will leak out of the mitochondria if the membrane potential is lost. Viable cells could be seen with intact mitochondria (DR staining) after 30 min of rehydration. After 60 min, viable cells were observed to reattach to the glass imaging plate lightly (differential interference contrast; DIC), and MVB relocalized to the mitochondria. Fluorescence intensities are not comparable between images.

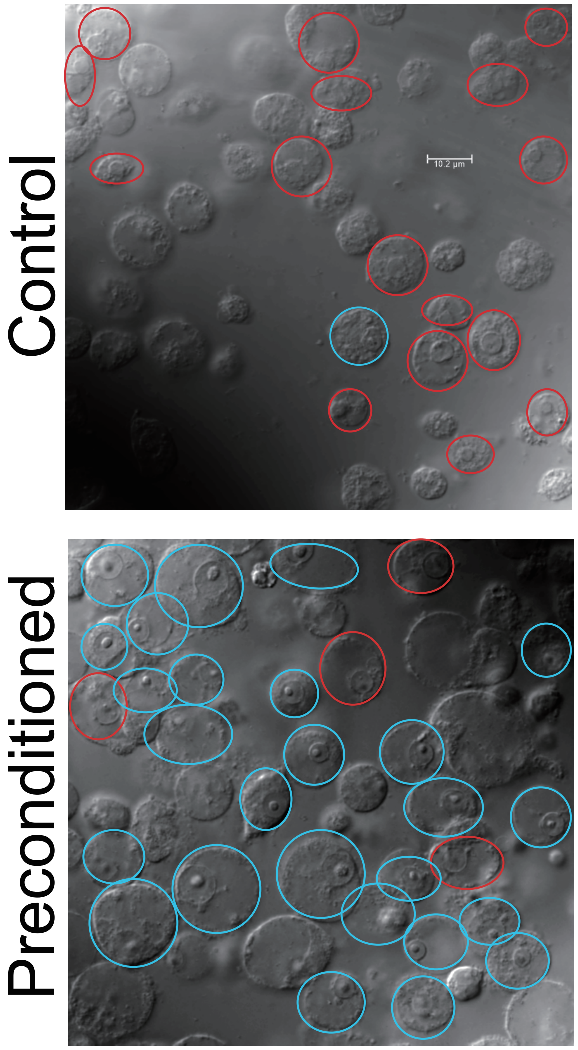

	Control	Preconditioned
-Nucleolus	17	4
+Nucleolus	1	26
Total Cells	17	30
% + Nucleolus	5.88	86.67

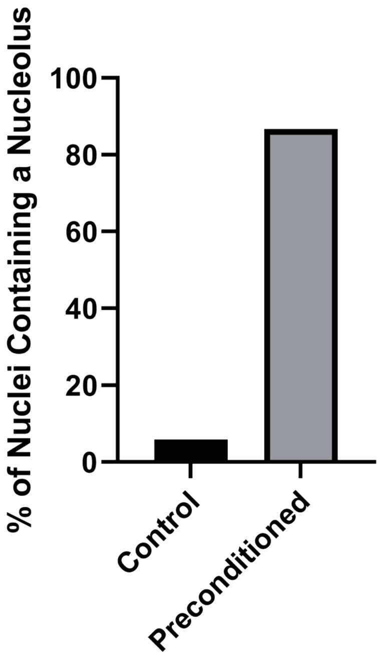

Fig. S3. The nucleolus is stabilized in ~86% of preconditioned Pv11 cells and only in ~6% of control cells. Confocal images are representative images that contain the rehydrated cells depicted in Fig. 4. Only cells with a visible nuclear envelope in the images were evaluated. Blue circles represent cells with a visible nucleolus, and red circles represent cells with no visible nucleolus.

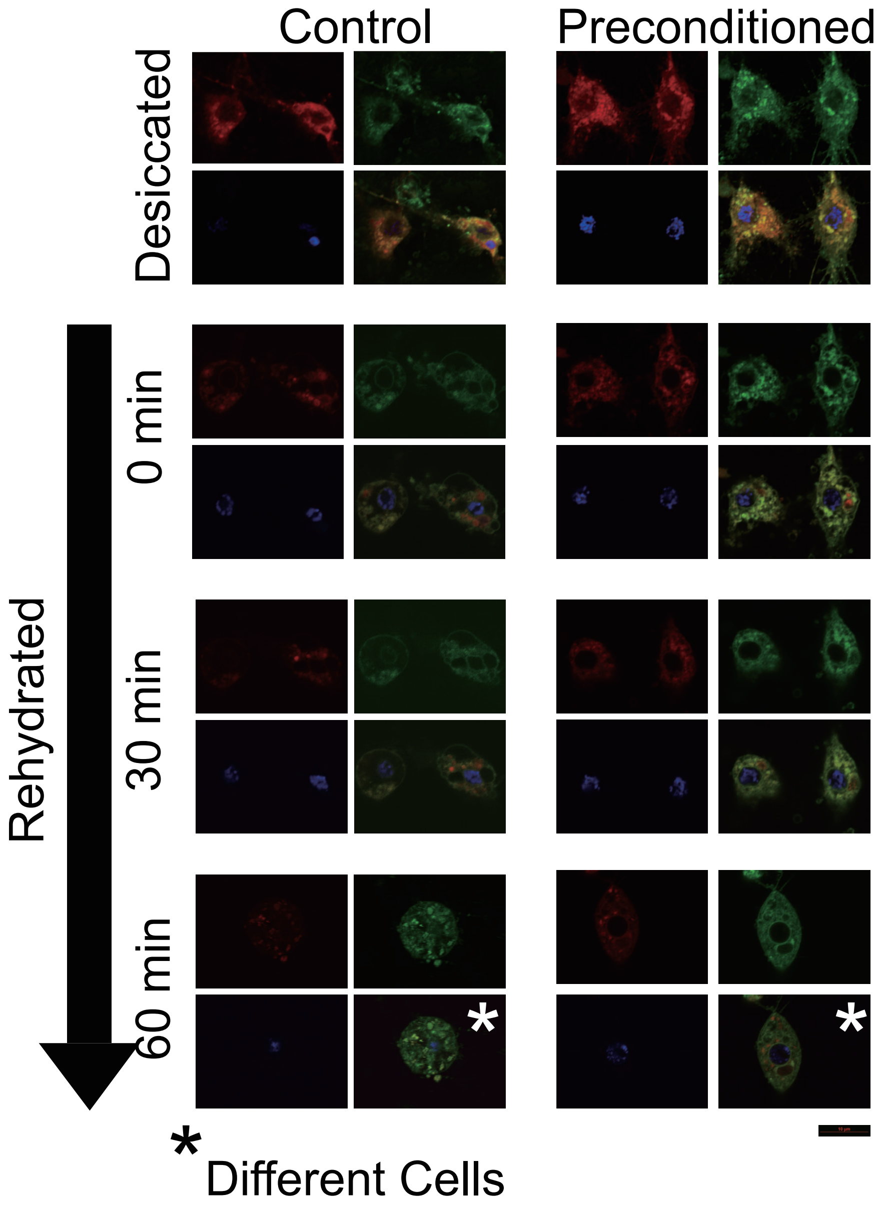

Fig. S4. Pv11 cells in culture can exhibit axon-like structures at one or both polar ends of their cell body. The ER/Golgi Cytopainter staining kit was used to stain the ER (red/orange), Golgi apparatus (green), and nucleus (blue). The red ER stain appears orange in merged images due to slight off-staining from the green Golgi apparatus stain. These structures contain an extended ER network (orange) and Golgi outposts (green nodes), both characteristic of neurons.

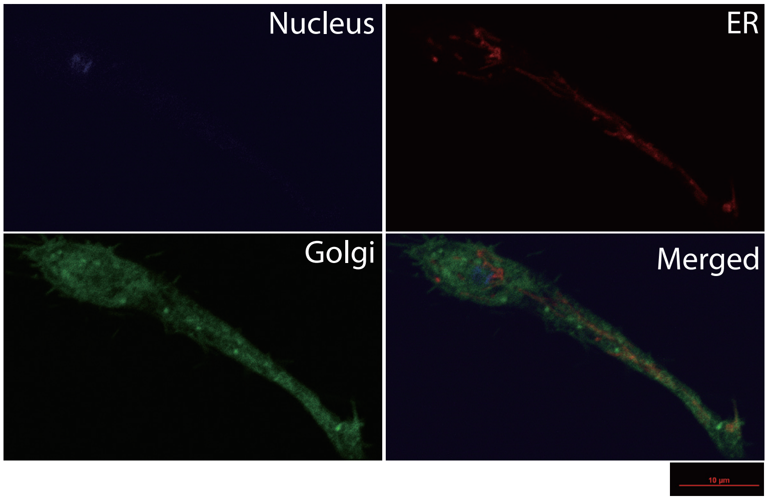

Fig. S5. Individual fluorescence channels for Fig. 6. The ER/Golgi Cytopainter staining kit was used to stain the ER (red/orange), Golgi apparatus (green), nucleus (blue), and the preconditioning-induced MLO (red). The red ER stain appears orange in merged images due to slight off-staining from the green Golgi apparatus stain. Recovery of the ER and Golgi apparatus was observed in preconditioned cells within 1 h following rehydration. The preconditioning-induced membraneless organelle dissociates upon desiccation but rapidly reassembles within 30 min after rehydration and progressively excludes the green Golgi apparatus stain. The orange and green staining observed in control cells results from unspecific staining due to loss of cellular organization. Fluorescence intensities are not comparable among images. Please see Fig. 6 for larger merged images.

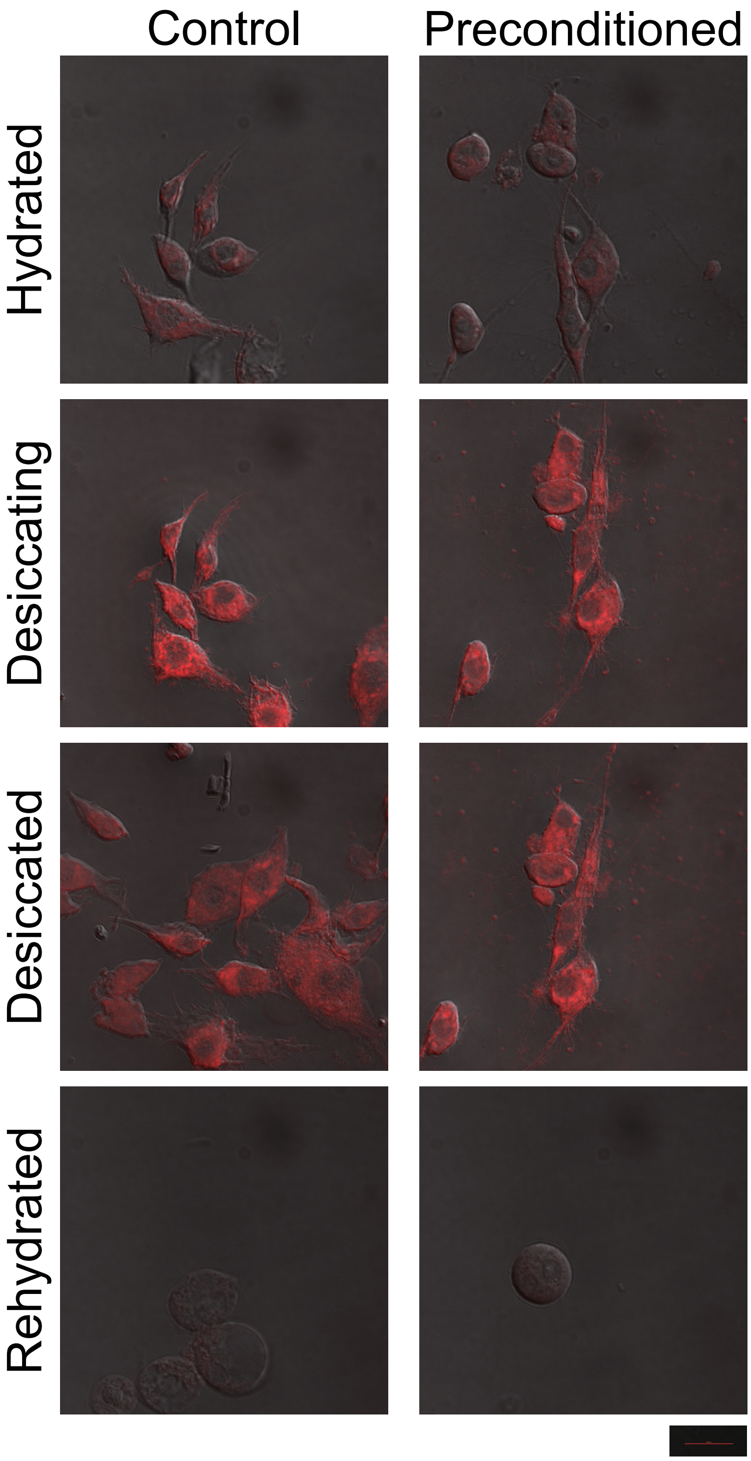

Fig. S6. Intracellular viscosity during desiccation is not significantly increased due to preconditioning. Nile Red (red), a solvatochromic dye, revealed no significant increase in intracellular viscosity between control and preconditioned Pv11 cells during desiccation. Fluorescence intensity is relative among all images. Cells in the rehydrated images are different cells. The scale bar represents 10 μm.
